# Trueness of Complete-Arch Implant Scans: Influence of Scanning Technique, Intraoral Scanner, and Implant Position (Part I: Linear and Angular Deviations)

**DOI:** 10.3390/medicina61122127

**Published:** 2025-11-28

**Authors:** Bersu Bedirhandede, Ayşe Demir Canbulut, Emre Tokar, Merve Çakırbay Tanış, Nihal Tokar

**Affiliations:** 1 Department of Prosthodontics, Faculty of Dentistry, Gazi University, Ankara 06560, Türkiye; ayse.demir6@gazi.edu.tr (A.D.C.); emretokar@gazi.edu.tr (E.T.); mervecakirbay@gazi.edu.tr (M.Ç.T.); 2 N-Dent Oral and Dental Health Center, Ankara 06130, Türkiye; dtnihalpehlivan@gmail.com

**Keywords:** digital impression, implant, intraoral scanner, trueness, scanning technique

## Abstract

*Background and Objectives*: This study aimed to investigate the effect of the intraoral scanner, scanning technique, and implant position on the trueness measured by linear and angular deviations. *Materials and Methods*: An edentulous maxillary model with four implants was scanned using four intraoral scanners (Trios 5, Trios 3, Primescan, Medit i700) and four scanning techniques (unmodified, composite, eugenol, dental floss). Each intraoral scanner–scanning technique combination was repeated ten times, producing 160 test datasets. Master reference files were generated with a laboratory scanner. Linear and angular deviations were calculated after superimposing each test scan with its master file. A three-way ANOVA followed by Tukey’s HSD test was used to determine statistical differences. Significance was set at *p* < 0.05. *Results*: Significant effects of intraoral scanner, scanning technique, and implant position were found for both linear and angular deviations (*p* < 0.001). Trios 5 showed the highest linear deviation values, although these remained within clinically acceptable limits, while Primescan showed the lowest. Dental floss produced the highest linear and angular deviations, whereas eugenol demonstrated the lowest. Medit i700 demonstrated the lowest angular deviation. *Conclusions*: All intraoral scanners showed deviations within clinically acceptable thresholds, although Trios 5 showed the highest linear deviation. Among scanning techniques, dental floss resulted in the highest linear and angular deviations. Deviations were lower between adjacent implants and higher across 1–3, 1–4, and 2–4.

## 1. Introduction

Computer-assisted design and computer-assisted manufacturing (CAD-CAM) is increasingly used in implant dentistry, and intraoral scanners (IOSs) are now an essential part of this workflow, offering accuracy comparable to conventional implant impressions. Their use for digital implant impressions has grown together with the routine application of intraoral scan bodies [1]. Today’s IOSs rely on several optical principles, including triangulation (active or passive), confocal imaging, active wavefront sampling, and optical coherence tomography. In triangulation, three-dimensional (3D) surface points are calculated from the geometric relationship between projected light and the sensor’s line of sight systems and a pinhole is applied to admit only in-focus reflected light, which enhances image contrast and resolution. Optical coherence tomography splits a low-coherence light source into reference and sample paths and uses interferometry to generate depth-resolved, high-resolution images and wavefront sampling projects with a changing light pattern and analyzes its deformation on surfaces to reconstruct accurate 3D models [2,3].

Although IOSs are widely used in clinical practice, the accuracy of digital complete-arch implant impressions is still considered inadequate [1,4,5,6]. Edentulous spans provide only a few stable landmarks, which makes the registration of sequential images more challenging and increases the risk of distortion during scanning [1,6,7]. In addition, IOSs generate a complete-arch dataset by continuously stitching multiple images, and minor misalignments in each step accumulate as the scanning distance increases [8]. These challenges have led to the development of techniques aimed at improving the accuracy of complete-arch digital implant scans.

Different techniques have been proposed to improve the accuracy of complete-arch implant scans, including directly or indirectly splinting the intraoral scan bodies and providing additional reference points [6,9]. These include adding reference geometry with artificial markers such as glass beads or zinc oxide–eugenol paste, and splinting scan bodies using dental floss, orthodontic wire, thermoplastic resin, or composite resin. Other reported methods are the use of temporary implant-supported prostheses, polymethyl methacrylate plates or bars, and 3D-printed guides with tooth-like or geometric features, as well as horizontal scan bodies with extension arms [4,9,10,11,12,13]. Although various techniques have been introduced to improve complete-arch implant scan accuracy, studies that evaluate how different IOSs and scanning techniques together affect scan trueness are still limited. Determining the trueness of digital scans obtained with different IOS and scanning technique combinations would help clinicians choose the most reliable and efficient method for scanning fully edentulous arches.

The purpose of the present study was to evaluate the influence of IOS, scanning technique, and implant position on the trueness of complete-arch intraoral implant scans. The null hypothesis was that different IOSs, scanning techniques, and implant positions would not affect the trueness of scans based on the linear distance deviation and angular deviation.

## 2. Materials and Methods

**Figure 1:**
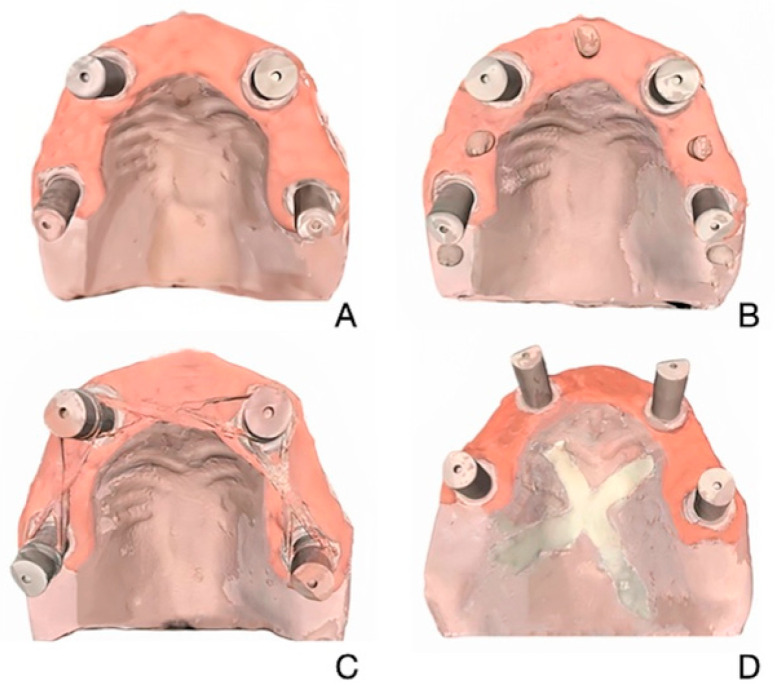
Edentulous maxillary model scanned with 4 scanning techniques. (**A**) Unmodified. (**B**) Composite. (**C**) Dental Floss. (**D**) Eugenol.

In this present study, four intraoral scanners—Trios 5 (T5; 3Shape, Copenhagen, Denmark), Trios 3 (T3; 3Shape, Copenhagen, Denmark), Primescan (PS; Dentsply Sirona Inc., Bensheim, Germany), Medit i700 (M; Medit, Seoul, Republic of Korea)—and four different scanning techniques—unmodified (UM), composite resin (C), eugenol (E), and dental floss (DF)—were evaluated (Figure 1). Based on a power analysis effect size f = 0.50 (power = 95%, α = 0.05), ten scans (n = 10) were obtained for each IOS and scanning technique [4].

### 2.1. Master Model Preparation and Reference Scanning

The master model was 3D printed from a standard tessellation language (STL) file generated by scanning a prefabricated clear acrylic resin maxillary typodont with four implants (two straight and two distally tilted; Nobel Active RP 4.3 × 13 mm, Nobel Biocare AG, Zürich, Switzerland) simulating the all-on-4 concept and 4 multiunit abutments (2 straight, Multi-unit Abutment Plus Conical Connection RP 2.5 mm; Nobel Biocare AG, Zürich, Switzerland and 2 30° inclined multiunit abutments, 30° Multi-unit Abutment Plus Conical Connection RP 3.5 mm; Nobel Biocare AG, Zürich, Switzerland) using a laboratory scanner (Medit T710, Medit Corp., Seoul, Republic of Korea), as the translucent surface of the typodont caused light scattering and interfered with scan accuracy [14]. 3D printing was performed using a polymer printer (Elegoo Mars, Shenzhen, China) and model resin (Power Dent Model, 3BFAB Technology Inc., İstanbul, Türkiye). The master model included pink gingiva around the scan abutment area and was fitted with four abutment analogs. For the scanning techniques, a light-cured composite was placed between adjacent scan bodies as a small, rounded mass in the C technique; zinc oxide eugenol paste was applied onto the palatal surface as a thin X-shaped reference mark in the E technique; and a dental floss was positioned between the scan bodies in the DF technique. Following the application of each scanning technique (UM, C, E, F), the model was scanned using a laboratory scanner (Medit T710) equipped with blue-light technology, featuring four integrated 5-megapixel cameras and a scanning accuracy of 4 μm. Each scan was exported as an STL file, resulting in four separate STL files representing the model under each condition. These files were used as master reference files. The laboratory reference scanner was calibrated prior to every scan.

### 2.2. Test Scans with Different Intraoral Scanners and Scanning Techniques

A total of 160 test scans were obtained by scanning the master model ten times (n = 10) for each of the four IOSs and each of the four scanning techniques. All scans were performed by a single operator to ensure consistency. With the exception of T5, all IOSs were calibrated before each scanning session in accordance with the manufacturers’ instructions. T5 was operated using a 5-GHz wireless connection, as specified by the manufacturer. In cases where image acquisition was compromised by artifacts, overlapping structures, or visible distortions, the respective scan was repeated to ensure data reliability. Scans were conducted under consistent ambient lighting and within a controlled environment for temperature and humidity. The scan body positions were labeled 1 through 4 according to implant positions (1: right first molar; 2: right canine; 3: left canine; 4: left first molar). The scanning pattern followed a standardized sequence, beginning from the occlusal surfaces of implants 1 to 4, proceeding to the palatal surfaces, and concluding with the buccal surfaces [1]. The test scans were exported as STL files.

### 2.3. Linear and Angular Deviation Assessment

All STL datasets were imported into metrology software (Geomagic Control X 2018, 3D Systems Inc. Rock Hill, SC, USA) to evaluate linear and angular deviations. Each test scan was aligned with the corresponding master reference STL file obtained using the same scanning technique. An initial alignment was performed using the software’s pre-alignment function. Subsequently, a refined registration was carried out using the “reference best fit algorithm”, in which the scan bodies were excluded from the superimposition process. Only unchanged anatomic regions of the model, such as the palatal vault, alveolar ridges, and basal area, were utilized for alignment to minimize registration errors and to prevent the influence of scan body discrepancies on the measurement outcomes. A reference plane was defined by selecting three points on the upper surfaces of the scan bodies. Two additional planes were created at 2.5 mm and 5 mm above this plane to assist in axis determination [4,15]. Circles were drawn on these planes, and their center points were identified to define the central axes of the scan bodies. Using the point function, coordinates required for distance measurements were marked, and the axes for angular measurements were generated by connecting the corresponding center points. For each scan, Cartesian coordinates (*x*, *y*, *z*) of six scan bodies were exported in .txt format. For each implant, the center point was calculated as the vector difference between the coordinates of the upper and lower circles [15]. These coordinate values were then used to calculate interimplant distances according to the following formula:

$$D=\sqrt{{\left(x_{a}-x_{b}\right)}^{2}+{\left(y_{a}-y_{b}\right)}^{2}\begin{array}{c} +{\left(z_{a}-z_{b}\right)}^{2} \end{array}}$$

**Figure 2:**
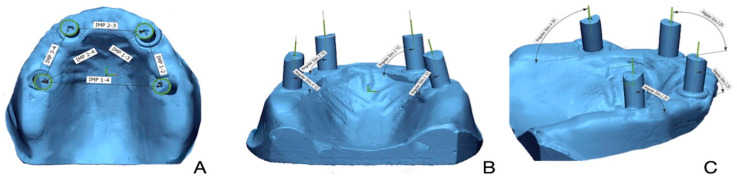
(**A**) Linear deviation measured between implant scan bodies. (**B**) Angular deviation measured on implant scan bodies with respect to YZ axis. (**C**) Angular deviation measured on implant scan bodies with respect to XZ axis.

Angular deviation was obtained by measuring the angle between the scan body axes of the test scan and the corresponding axes of the master reference scan after alignment in the metrology software. This comparison was performed separately relative to the XY and YZ planes to assess directional components of deviation (Figure 2).

### 2.4. Statistical Analysis

All statistical analyses were performed using SPSS software (version 21, IBM Software, Armonk, NY, USA) with a significance level set at *p* < 0.05. Normality of data distribution was confirmed using the Kolmogorov–Smirnov and Shapiro–Wilk tests. Three-way ANOVA was used to assess the combined effects of IOS, scanning technique, and implant position. Post hoc analyses were conducted using Tukey’s HSD test.

## 3. Results

Linear and angular deviation results from the three-way ANOVA are shown in Table 1. Both parameters differed significantly according to IOS, scanning technique, and implant position (*p* < 0.001). Significant two-way interactions were observed between IOS and implant position, and between IOS and scanning technique (*p* < 0.001). Implant position–scanning technique interaction was also significant for linear deviation (*p* = 0.003) and for angular deviation (*p* < 0.001). A significant three-way interaction among all factors was found for both linear and angular deviations (*p* < 0.001).

**Figure 3:**
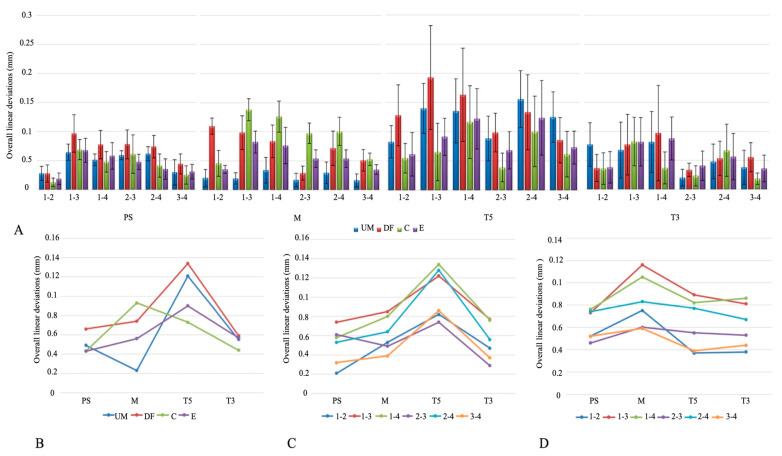
(**A**) Overall linear deviations. (**B**) Intraoral scanner × scanning technique interaction plot. (**C**) Intraoral scanner × implant position interaction plot. (**D**) Scanning technique × implant position interaction plot. Abbreviations: PS—Primescan; M—Medit; T5—Trios5; T3—Trios3; UM—unmodified; DF—dental floss; C—composite; E—eugenol.

The overall linear deviations are shown in Figure 3A. T5 had the highest mean linear deviation, significantly greater than M, T3, and PS (*p* < 0.001), while PS showed the lowest values, with no difference between T3 and either M or PS (*p* > 0.05). Deviations were lowest at positions 3–4, 1–2, and 2–3, and significantly higher at 1–3, 1–4, and 2–4 (*p* < 0.001). Among scanning techniques, DF produced the greatest deviation compared with UM, C, and E (*p* < 0.001), which did not differ from each other (Table 2).

Analysis of the interaction between IOSs and scanning techniques showed significant differences in mean linear deviation for PS, M, and T5 (*p* < 0.05), but not for T3 (*p* > 0.05). For PS, the DF technique produced the highest values; for M, the C technique showed the largest deviations; and for T5, both UM and DF were highest (Figure 3B). IOS–implant position interaction also revealed significant differences (*p* < 0.05). In all IOSs, deviations at positions 1–3, 1–4, and 2–4 were higher than at 1–2, 2–3, and 3–4 (Figure 3C). Scanning technique–implant position interaction further indicated that 1–3 and 1–4 consistently showed the greatest deviations across techniques, while 2–3 and 2–4 were also elevated in UM, DF, and E, and in the C technique, higher values were noted at 1–3, 1–4, and 2–4 (Figure 3D).

**Figure 4:**
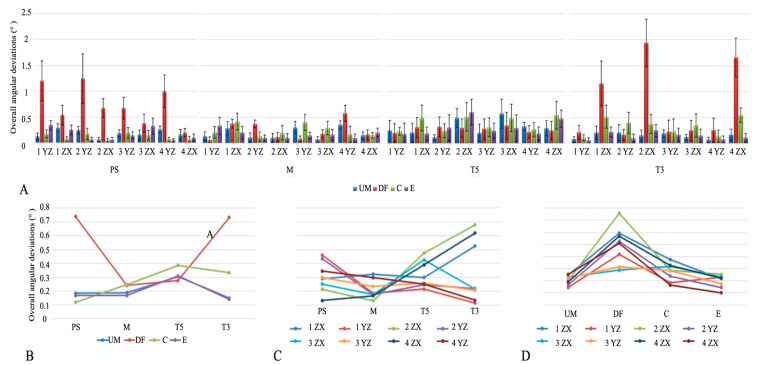
(**A**) Overall angular discrepancies. (**B**) Intraoral scanner × scanning technique interaction plot. (**C**) Intraoral scanner × implant position interaction plot. (**D**) Scanning technique × implant position interaction plot. Abbreviations: PS—Primescan; M—Medit; T5—Trios5; T3—Trios3, UM—unmodified; DF—dental floss; C—composite; E—eugenol.

The overall angular deviations are shown in Figure 4A. Angular deviation was significantly lower with M compared to T3 (*p* < 0.001), while PS and T5 did not differ from either. By implant position, the YZ axis of implant 1 showed the lowest deviation, whereas higher values were recorded at the XZ axes of implants 1, 2, and 4 (*p* < 0.001). Among scanning techniques, the E technique produced the lowest angular deviation, and DF showed the highest (*p* < 0.001, Table 3).

The interaction between IOSs and scanning techniques showed significant differences in angular deviation (*p* < 0.05). DF produced the highest values in PS and T3, while in M, both DF and C were higher than E, and in T5, the greatest deviation was seen with C (Figure 4B). IOS–implant position interaction also revealed significant differences (*p* < 0.05). In PS, higher deviations occurred at the YZ axes of implants 1, 2, and 4; in M, at the XZ axis of implant 1 and YZ axis of implant 4; in T5, at the XZ axes of implants 2, 3, and 4; and in T3, at the XZ axes of implants 1, 2, and 4 (Figure 4C). Scanning technique–implant position interaction showed significant differences for all techniques except UM. In DF, the XZ axis of implant 2 showed the highest deviation (*p* < 0.05). In C, greater deviations were noted at the XZ axes of implants 1–4 and the YZ axis of implant 3, while in E, the XZ axes of implants 2–4 and the YZ axis of implant 1 were higher compared to the YZ axis of implant 4 (*p* < 0.05, Figure 4D).

## 4. Discussion

Based on the results, the null hypothesis was rejected, as different IOSs, scanning techniques, and implant positions had significant effects on the trueness of scans based on linear distance deviation and angular deviation.

Reported trueness for digital complete-arch implant impressions ranges from 0.032 to 0.452 mm for linear distance deviation [4,6,8,9,10,16,17] and from 0.13° to 0.34° for angular deviation [9,10,16]. Consistent with these ranges, the present study showed linear deviations of 0.050, 0.061, 0.104, and 0.053 mm and angular deviations of 0.30°, 0.21°, 0.31°, and 0.33° for PS, M, T5, and T3, respectively. Considering 0.100 mm and 0.4° as clinically acceptable thresholds, the findings demonstrated that the deviations were generally within these limits [18]. As physical models or frameworks were not fabricated from the digital implant impressions in this study, avoiding these additional laboratory steps likely kept overall deviations below or near 0.100 mm, while the T5 condition slightly exceeded this level. In routine clinical workflows, inclusion of model and framework fabrication can introduce additional sources of error, and total deviations may exceed 0.100 mm [19].

For complete edentulous maxilla with five implants Revilla-León et al. [16] reported that T5 (0.050 mm), PS (0.052 mm), and Aoralscan 3 (0.053 mm) showed significantly better linear deviation than M (0.011 mm), and that angular deviation means were T5 (0.41°), Aoralscan 3 (0.29°), PS (0.48°), M (0.35°), and iTero (0.44°), indicating the best angular trueness for Aoralscan 3 and M. Also, Grande et al. [7] found that the T3 scanner showed the lowest linear deviation (0.085 mm), while the angular deviation values were 0.28° for T3, 0.17° for M, 0.23° for iTero Element 5D, and 0.23° for PS, indicating that the M scanner exhibited the lowest angular deviation. Additionally, an in vitro comparison of digital acquisition methods for the reverse impression technique reported that T3 showed a linear deviation of 0.011 mm, T5 of 0.085 mm, PS of 0.010 mm, and M of 0.012 mm, with T5 showing less linear deviation than PS, while no significant differences were found between T5, T3, and M [20]. In the present study, T5 exhibited the highest mean linear deviation, significantly greater than M, T3, and PS, whereas angular deviation was lowest with M, which is consistent with previous studies, while the linear deviation result differs. Several factors may explain the higher linear deviation observed with T5. The absence of a clearly defined scanning protocol from the manufacturer may have increased operator-related variability, and because the literature provides limited guidance on the optimal strategy for complete-arch implant impressions, the same protocol was applied to all IOSs in this study [21]. The device design may also play a role; the small and wireless camera of T5 allows easier handling, but this mobility can introduce minor hand movements that increase the risk of stitching or matching errors [22]. Moreover, wireless data transfer at 5 GHz requires a stable connection, and issues such as signal interference, bandwidth limitations, or brief connection loss may compromise the point cloud. Such interruptions can affect the reconstruction of the 3D mesh and lead to reduced accuracy or incomplete surface [22,23]. Additional differences may further result from variations in software, scanner technology, acquisition strategies, and operator experience.

Previous studies show that IOS-based impressions for single implants provide clinically acceptable trueness and chairside efficiency. As indications expand to multiple implants, especially in completely edentulous arches, the trueness required increases and the procedure becomes more complex; digital workflows are reliable for single crowns and short-span fixed partial dentures, whereas complete-arch prostheses still tend to be less accurate [19,24,25]. Completely edentulous arches make stitching in IOS reconstructions difficult. IOS combines many small images, but these arches have few stable landmarks, long spans of movable mucosa, and multiple identical scan bodies. The gaps between scan bodies lack reference points and create minor vertical steps, making alignment less reliable. These factors together increase the chances of mis-stitching and lead to greater deviations [12,19]. In maxillary scans, the hard palate may act as a stabilizing landmark because its rigid vault and rugae provide fixed geometric features that could assist stitching continuity [19]. However, Mizumoto et al. [26] reported that including or excluding the palate did not significantly affect trueness in the maxilla, suggesting that its influence is scanner-dependent. Additionally, optical factors such as surface reflectivity or residual salivary film may further influence whether the palate facilitates or complicates data acquisition. Proposed solutions focus on adding reference geometry and connecting scan bodies to improve stitching and image alignment. Methods include placing artificial markers such as glass beads, marking the mucosa with pressure-indicating paste mixed with zinc-eugenol cement, and splinting scan bodies with dental floss, orthodontic wire, thermoplastic resin, or light-cured composite. Other options are using a temporary implant-supported prosthesis, PMMA arch-shaped plates or bars, 3D-printed arch-shaped guides with tooth-like features between scan bodies, 3D-printed guides with geometric shapes, and horizontal scan bodies with extension arms that contact the adjacent unit. Together, these adjuncts add distinctive references, stabilize registration, and reduce cumulative stitching error in full-arch scans [4,9,10,11,12,13]. In the present study, considering the complexity and time demands of many published techniques, three chairside approaches were evaluated, consisting of placing resin between adjacent scan bodies, drawing opaque reference lines with zinc oxide eugenol [27,28] on the palatal surface, and splinting scan bodies with dental floss [4]. Among these dental techniques, DF groups exhibited the highest linear and angular deviation. These results align with Kanjanasavitree et al. [25], who found that direct DF contact with the scan-body surface can impair IOS surface capture. Similarly, Kalaycı et al. [29] reported that splinting scan bodies with flexible auxiliary markers did not improve accuracy, likely due to insufficient rigidity and continuity of the splint material, which disrupts stitching and reduces trueness. Mizumoto et al. likewise observed the highest distance deviation with the DF technique, indicating that splinting scan bodies intended to improve stitching does not enhance accuracy [4]. Additionally, with implant splinting, increased shadowing under the splint material extending between adjacent implants may be associated with reduced scanning efficiency [9]. The present study also revealed that DF resulted in the highest linear deviation across all scanners, whereas C produced the highest deviation specifically with the M. These findings indicate that the underlying scanning technology may influence trueness. T5, T3, and PS are based on confocal microscopy, while M functions with triangulation principles [2]. Triangulation partly relies on the geometry and surface characteristics of the object [30] and the irregular form of the applied resin may therefore account for the greater deviation observed in the composite group with M. In active triangulation, images are captured from two different perspectives and combined into a 3D model through trigonometric calculations of the X, Y, and Z coordinates. In contrast, confocal microscopy collects a series of focused images using a larger optical receiver. This requirement makes it more difficult to scan objects in close proximity to the scanner tip, as light emission can be hindered [31].

Regarding the effect of implant position on trueness, deviations measured between adjacent implants (1–2, 2–3, 3–4) were lower compared with those spanning longer distances (1–3, 1–4, 2–4). Comparable outcomes were reported by Mizumoto et al. [26], who identified significant differences in distance deviation depending on cross-arch scan body locations (1–3, 1–4, 2–3, 2–4), regardless of whether the scan bodies were placed anteriorly or posteriorly, and related this finding to the curvature of the maxillary arch. Çakmak et al. [32] also demonstrated that distance deviations varied significantly according to scan body position across the arch. Consistently, Schmit et al. [3] found the highest deviation between the two molar positions (1–4), supporting the tendency for larger deviations in posterior regions. This increase in deviation posteriorly can be explained by superimposition or matching error. When recording begins from the upper right maxilla, small inaccuracies from each step accumulate as the process continues, and the greatest mismatches appear at the furthest point [3,26,33]. In the present study, implant positions were further characterized by larger angular deviations on the XZ plane. When this angular deviation is considered together with the increased linear deviation observed between implants 1–4, the results indicate a transverse expansion of scan data in the posterior edentulous arch [34]. Such angular changes in the XZ plane correspond to rotation around the *y*-axis (mesiodistal), and this relationship helps explain the potential clinical impact. Restorations derived from these scans may present buccal or lingual overcontouring or undercontouring, which could require additional intraoral adjustments [23].

From a clinical standpoint, deviations in full-arch digital scans may influence the fit and behavior of the prosthetic components in several ways. Angular discrepancies can affect screw–abutment seating and contribute to torque loss, while linear deviations may create localized binding points during framework placement. On longer edentulous spans, cumulative errors can alter occlusal relationships or internal stress distribution within the superstructure, potentially affecting long-term mechanical and biological outcomes.

As an in vitro study, clinical factors such as saliva, moisture, light, and patient movement were not reproduced, potentially influencing trueness [11,31,32,33]. Scanning plaster models is another limitation, as they are easier to capture than intraoral conditions with limited space and complex optical properties. The reference model was digitized with a 5 μm desktop scanner, which is widely used but still may contain possible errors [5]. Only one wireless IOS was tested, scan bodies were connected to analogs rather than abutments, and a single operator used a scan path not validated for edentulous arches, which may affect outcomes [4]. Future in vivo studies should evaluate how the deviations observed in vitro behave under real intraoral conditions, where saliva, soft-tissue mobility, and patient movement may influence scan accuracy. Such investigations would help confirm the clinical applicability of different scanners and scanning techniques for full-arch implant rehabilitation.

## 5. Conclusions

This in vitro study demonstrates that IOS selection, scanning technique, and implant position significantly affect the trueness of complete-arch digital implant impressions. Although the T5 condition showed the highest linear deviation among all measurements, the values remained within clinically acceptable limits, confirming that the evaluated scanners are suitable for implant-supported prostheses. The DF technique, on the other hand, led to significantly higher deviations, indicating that it should be avoided in clinical applications. Furthermore, deviations increased with longer interimplant distances (1–3, 1–4, 2–4) compared to adjacent pairs (1–2, 2–3, 3–4), reflecting the cumulative effects of stitching and matching errors across the arch. Overall, these results offer evidence-based recommendations for optimizing scanner and protocol selection in implant prosthodontics, suggesting that while any of the tested scanners can be reliably used for complete-arch cases, clinicians should consider the negative impact of the dental floss technique on trueness.

## Figures and Tables

**Table 1:** Three-way ANOVA results for linear and angular deviation, considering the factors intraoral scanner, implant position, and scanning technique.

Linear Deviation				Angular Deviation			
Factors	df	F	*p*	Factors	df	F	*p*
IOS	3	130.62	<0.001	IOS	3	37.61	<0.001
IP	5	49.17	<0.001	IP	7	15.95	<0.001
ST	3	22.68	<0.001	ST	3	229.68	<0.001
IOS × IP	15	4.60	<0.001	IOS × IP	21	33.04	<0.001
IOS × ST	9	24.34	<0.001	IOS × ST	9	85.17	<0.001
IP × ST	15	2.30	0.003	IP × ST	21	10.92	<0.001
IOS × IP × ST	45	2.68	<0.001	IOS × IP × ST	63	19.19	<0.001

IOS—intraoral scanner; IP—implant position; ST—scanning technique.

**Table 2:** Mean and standard deviation (mm) for the pairwise comparisons of linear deviation.

Factor		Mean ± Standard Deviation	*p*
IOS	PS	0.05 ± 0.03 ^a^	<0.001
	M	0.06 ± 0.04 ^b^	
	T5	0.10 ± 0.06 ^c^	
	T3	0.05 ± 0.04 ^ab^	
IP	1–2	0.05 ± 0.04 ^a^	<0.001
	1–3	0.09 ± 0.05 ^b^	
	1–4	0.09 ± 0.06 ^b^	
	2–3	0.05 ± 0.03 ^a^	
	2–4	0.08 ± 0.05 ^c^	
	3–4	0.05 ± 0.04 ^a^	
ST	UM	0.06 ± 0.05 ^a^	<0.001
	DF	0.08 ± 0.06 ^b^	
	C	0.06 ± 0.05 ^a^	
	E	0.06 ± 0.04 ^a^	

Different superscript lowercase letters denote significant differences. Abbreviations: IOS—intraoral scanner; IP—implant position; ST—scanning technique; PS—Primescan; M—Medit; T5—Trios 5; T3—Trios 3; UM—unmodified; DF—dental floss; C—composite; E—eugenol.

**Table 3:** Mean and standard deviation (°) for the pairwise comparisons of angular deviation.

Factor		Mean ± Standard Deviation	*p*
IOS	PS	0.30 ± 0.35 ^a^	<0.001
	M	0.21 ± 0.16 ^c^	
	T5	0.32 ± 0.22 ^ab^	
	T3	0.34 ± 0.46 ^b^	
IP	1 YZ	0.24 ± 0.30 ^a^	<0.001
	1 XZ	0.36 ± 0.30 ^b^	
	2 YZ	0.27 ± 0.32 ^a^	
	2 XZ	0.37 ± 0.48 ^b^	
	3 YZ	0.25 ± 0.20 ^a^	
	3 XZ	0.27 ± 0.22 ^a^	
	4 YZ	0.26 ± 0.27 ^a^	
	4 XZ	0.33 ± 0.40 ^b^	
ST	UM	0.21 ± 0.16 ^a^	<0.001
	DF	0.50 ± 0.51 ^c^	
	C	0.27 ± 0.21 ^b^	
	E	0.20 ± 0.17 ^a^	

Different superscript lowercase letters denote significant differences. Abbreviations: IOS—intraoral scanner; IP—implant position; ST—scanning technique; PS—Primescan; M—Medit; T5—Trios 5; T3—Trios 3; UM—unmodified; DF—dental floss; C—composite; E—eugenol.

## Data Availability

The data relating to this study are available upon reasonable request from the corresponding author.

## Abbreviations

The following abbreviations are used in this manuscript:

CAD-CAM	Computer-assisted design and computer-assisted manufacturing
IOS	Intraoral scanner
T5	Trios 5
T3	Trios 3
M	Medit
PS	Primescan
UN	Unmodified
C	Composite
E	Eugenol
DF	Dental floss
STL	Standard tessellation language
IP	Implant position
ST	Scanning technique
